# Model systems for emulating human tissue and physiology in psychiatric research

**DOI:** 10.3389/fnins.2025.1527826

**Published:** 2025-04-04

**Authors:** Kai Cheng, Anannya Kshirsagar, John Nixon, Jonathan Lau, Kun Yang, Akira Sawa, Annie Kathuria

**Affiliations:** ^1^Department of Biomedical Engineering, Whiting School of Engineering, Johns Hopkins University, Baltimore, MD, United States; ^2^Department of Psychiatry and Behavioral Sciences, Johns Hopkins School of Medicine, Baltimore, MD, United States; ^3^Departments of Psychiatry, Neuroscience, Mental Health, Pharmacology, Biomedical Engineering, and Genetic Medicine, Johns Hopkins University School of Medicine and Bloomberg School of Public Health, Johns Hopkins Medicine, Baltimore, MD, United States; ^4^Kavli Neuroscience Discovery Institute, Johns Hopkins University, Baltimore, MD, United States

**Keywords:** 3D organoid models, iPSC, tissue engineering, regenerative medicine, computational modeling

## Abstract

The modeling of psychiatric disorders poses significant challenges due to the complex nature of these conditions, which encompass a range of neuropsychiatric diseases such as autism spectrum disorder (ASD), schizophrenia (SCZ), bipolar disorder (BD), post-traumatic stress disorder (PTSD), anxiety disorder (AD) and depression. The rising global prevalence of mental disorders and the urgency for more effective treatments have propelled the development of innovative *in vitro* models. This review presents a thorough examination of two-dimensional (2D) versus three-dimensional (3D) induced pluripotent stem cell (iPSC) models of neuropsychiatric diseases, offering insights into their respective capacities to mimic neurodevelopment and cellular phenotypes observed in these conditions. Our comparative analysis reveals that while traditional 2D cultures have been instrumental in elucidating disease pathways and high-throughput drug screening, they fall short in replicating the intricate cellular architecture and environment of the human brain. On the other hand, 3D organoid models, including brain organoids, better recapitulate the spatial organization, cell-type diversity, and functional connectivity of brain tissue, offering a more physiologically relevant context for studying disease mechanisms and testing therapeutic interventions. We assess the progress in modeling ASD, SCZ, BD, PTSD, AD, and depression, highlighting the advanced understanding of disease etiology and potential treatment avenues offered by 3D iPSC technologies. Challenges remain, including the scalability, reproducibility, and maturation of organoids, but the potential for personalized medicine and the elucidation of disease ontogeny is unparalleled. The review concludes with a perspective on the future directions of psychiatric disease modeling, emphasizing the integration of 3D iPSC models with high-throughput technologies and computational approaches to enhance our understanding and treatment of these debilitating conditions.

## Graphical abstract

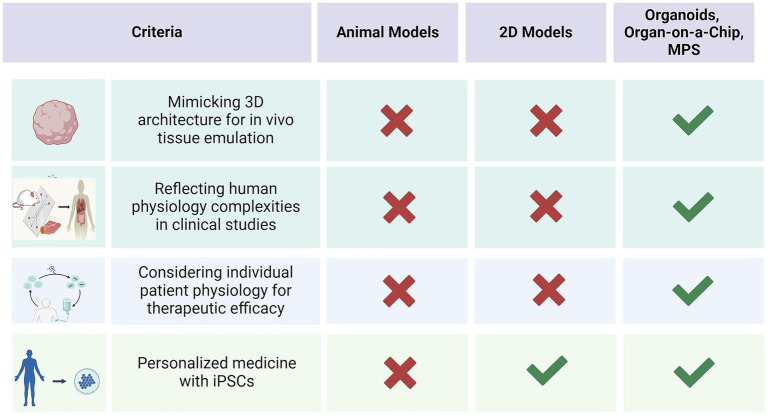

Experimental models differ (Animal Models, 2D Models, and Organoids/Organ-on-a-Chip/MPS) based on four key criteria for disease modeling and therapeutic development: emulating 3d tissue architecture and human physiological complexity, taking into consideration any individual patient physiology, and capacity for personalized medicine using iPSCs; these criteria indicate the relative strengths (green checkmarks) and limitations (red X's) of each approach.

## Introduction

### Need for modeling psychiatric disorders

Psychiatric disorders are common worldwide. According to the World Health Organization (WHO), one in every eight people around the world is living with mental disorders, with anxiety and depressive disorders being the most common. Mental disorders affect one in every four people at some point in their lives and have become one of the leading causes of death globally since the significant rise of anxiety and depressive disorders due to the COVID-19 pandemic (World Health Organization, 2022; Institute of Health Metrics and Evaluation, 2022). Understanding the neurodevelopment and cellular context, organoids have emerged as a promising model system in the field.

### Limitations of animal models

Animal models are used to study diseases and their pathways. These models are based on the assumption that the pathways involved in the disease are conserved across different species, which means that the same genes and proteins are involved in the disease in both humans and animals. However, this assumption is not always true, and there may be differences in the pathways between species. Animal model relies on evolutionary conservation of pathway, limited by their inability to recreate human-specific processes. This is because there are many unique processes that contribute to brain development in humans that are not present in animals. For example, the development of the human cerebral cortex is much more complex than that of other animals. Although tools like cognitive bias testing are widely used to evaluate behaviors related to anxiety, depression, and pain in animals, they fail to capture hallmark symptoms of neuropsychiatric disorders, such as hallucinations, guilt, and delusions. These symptoms are inherently human and cannot be replicated in animal models. As a result, the conclusions drawn from such models may lack relevance to human conditions. Therefore, animal models may not accurately reflect the processes involved in human brain development and disease. This can lead to inaccurate conclusions and hinder the development of effective treatments for human diseases.

## 2D models of psychiatric disorders

2D models utilizing human pluripotent stem cells (hPSCs) offer a human-specific framework for investigating neurodevelopmental and psychiatric disorders. These conditions are difficult to analyze using conventional animal models, due to their subjective symptoms and intricate genetic complex backgrounds. Further, patient-derived hPSCs retain the individual’s genetic profile, allowing researchers to understand how genetic factors contribute to disease phenotypes. 2D hPSC models have been used to better understand the underlying cellular and molecular mechanisms that underlie a variety of pyschiatric disorders including autism spectrum disorder (ASD), schizophrenia (SCZ), bipolar disorder (BD), post-traumatic stress disorder (PTSD), and major depressive disorder (MDD) (Stern et al., 2018), as summarized in Table 1.

**Table 1:** Summary of cellular phenotypes of different disorders in 2D models.

Disease	Cell type	Region	Observations/Findings	References
MDD	Cortical Neurons	Cortex	Mushroom spine dendrites have greatly elevated length in cell lines that respond to bupropion treatment. Non-responsive lines have a non-significant reduction in dendrite length. In MDD cell lines derived from patients that did not respond to SSRIs, lowered expression of protocadherin-𝛼 genes resulted in altered neurite growth. Knockdown of protocadherin-𝛼 resulted in improved neurite length.	Avior et al. (2021) and Vadodaria et al. (2019)
MDD	Astrocytes	Cortex	Differential expression of genes related to GPCR ligand binding, synaptic signaling, and ion homeostasis.	Heard et al. (2021)
BD	NPCs	Cortex	Treatment of Li-responsive BD-NPCs with lithium showed increase in oxygen consumption and cellular respiration activity, while similar results were observed in non-responsive NPCs treated with valproate. Treatment of BD-NPCs with thapsigargin resulted in a decrease in store-operated calcium-release (SOCE) as measured by finding the AUC of Ca fluorescence assays. BD-NPCs display unique transcriptomic profile suggestive of accelerated differentiation.	Hewitt et al. (2023) and Osete et al. (2021)
BD	Neurons	Cortex	Differences in expression of genes related to focal adhesion, ECM, spliceosome, and oxidative phosphorylation between lithium-responsive and non-responsive iPSC-derived BD neurons.	Stern et al. (2018), Niemsiri et al. (2023), and Osete et al. (2021)
PTSD	Glutamatergic Neurons	HPA axis	Glucocorticoid regulation gene FKBP5 is differentially expressed in iPSC lines exposed to dexamethasone to simulate stress response. Heightened sensitivity to low levels of glucocorticoids is observed in iPSC lines derived from combat veterans experiencing PTSD, especially in mature neural cells as opposed to immature cells and NPCs.	Pernia et al. (2021) and Seah et al. (2022)
PTSD	Excitatory neurons	General	Differential expression of glucocorticoid signaling pathways in comparison with MDD	Chatzinakos et al. (2023)
SCZ	Cortical Interneurons	Cortex	Impaired mitochondrial function, reduced arborization, and decreased synapse formation and synaptic GABA release in SCZ cINs co-cultured with microglia. Notable reduction in synaptic puncta density (thought to be caused by lower level of NLGN2) and a decrease in action potential frequency, but both were rescued by treatment with N-Acetylcysteine.	Kathuria et al. (2019) and Park et al. (2020)
SCZ	Cortical Pyramidal Neurons	Cortex	Reduced dendritic spine density observed in iPSC-derived SCZ neurons compared to controls, in agreement with postmortem analysis of SCZ patients. Thought to be caused by differential expression of NRXN3 204 isoform in SCZ. Deficiencies in mitochondrial function and oxidative phosphorylation are observed in SCZ lines.	Kathuria et al. (2023) and Ni et al. (2020)
SCZ	Glial Cells (inc. astrocytes)	Cortex	miRNAs (miR-337-3p, miR-127-5p, miR-206, miR-1185-1-3p) exhibit highly reduced expression in SCZ-derived astrocytes compared to control lines. Altered calcium signaling, decreased glutamate uptake, and metalloproteinase activity observed in SCZ iPSC-derived glial cells. These differences are thought to contribute to altered inflammatory responses to IL-1𝛽.	Akkouh et al. (2020), Akkouh et al. (2021), and Szabo et al. (2021)
ASD	NPCs	General	Increased growth rate with poor-quality neural network formation deficient in synapses and with elevated DNA damage, and possible dysfunction in 𝛽-catenin/BRN2 signaling cascade. IGF-1 was found to rescue neuronal network formation defects via stimulation of 𝛽-catenin/BRN2 signaling.	Russo et al. (2018), Beopoulos et al. (2022), and Griesi-Oliveira et al. (2021)
ASD	Astrocytes	General	Low levels of complement c4 thought to contribute to impaired synaptic pruning. Blocking IL-6 secretion resulted in increased synaptogenesis of neurons in ASD-derived co cultures.	Wang et al. (2020) and Mansur et al. (2021)
ASD	Neurons	Cortex	Early iPSC study suggests implication of TRPC6 in alterations to dendritic spines and excitatory synapses. Significant decrease in synaptic gene expression and protein levels, glutamate neurotransmitter release, and a reduced spontaneous firing rate. Dysregulation of 20 genes related to synapses, dendrite elongation, and neurotransmitter release, as well as a significant decrease in dendrite length and number of ramifications.	Mansur et al. (2021), Griesi-Oliveira et al. (2015), and Griesi-Oliveira et al. (2021)
ASD	GABAergic neurons	Cortex	Dysregulation/imbalance of excitatory/inhibitory signaling due to lack of cadherin-13 in ASD-derived cells.	Wang et al. (2023)

Coculturing, which merges various cell types in a single culture system, can be used to investigate how different cell types influence each other, termed non-cell-autonomous interactions. It also allows for the examination of contact-dependent and independent cell interactions (Whiteley et al., 2022). This enhances 2D models by more accurately mimicking the underlying mechanisms of neuropsychiatric disorder pathogenesis. Cocultures do have certain limitations, in that they lack tissue architecture, cell-type diversity, dynamic growth expansion, and maturation.

The main benefit of 2D models compared to 3D organoid models lies in their simplicity, reproducibility, and scalability, enabling larger experiments and more extensive high-throughput screening. However, the limitations of 2D cell culture models, including their lack of tissue complexity and inability to replicate key *in vivo* conditions, significantly limit their effectiveness in elucidating the pathophysiology of psychiatric disorders. These challenges underscore the critical need for organoid models that offer a more physiologically relevant and complex platform for understanding the intricacies of psychiatric disease mechanisms.

### Autism spectrum disorder

iPSCs retain the unique genetic background of the individual which is paramount for studying ASD where roughly 80–85 percent of the population is idiopathic in origin. Research shows that neural cells derived from induced pluripotent stem cells (iPSCs), including both precursor cells and neurons that have undergone maturation, closely mimic the characteristics of human cortical cells during the embryonic stage, specifically between weeks 8–24. Therefore, these iPSC-derived neural cells serve as an ideal tool for investigating the initial stages of human neurodevelopment, especially when exploring genetically complex disorders such as ASD (Turkalj et al., 2020).

In general, aberrant neuronal maturation, varied neuronal differentiation, and synaptic formation have been implicated in iPSC models focused on APD (Mariani et al., 2015). Given the association between ASD and macrocephaly, excessive neural growth was also proposed to be associated with ASD. This has been demonstrated in iPSC models which showed increased neutral progenitor cell growth compared to controls. Additionally, this increased NPC growth in ASD individuals with macrocephaly was associated with an altered DNA replication program and increased DNA damage (Wang et al., 2020). Increased NPC growth was hypothesized to be due to the dysregulation of a beta-catenin/BRN2 transcriptional cascade. Neural networks formed from ASD-derived neuronal were found to be dysfunctional, characterized by abnormal neurogenesis and reduced synapse formation. Insulin growth factor 1, a drug in clinical trial for ASD, was able to rescue defects in this neural network, demonstrating the potential utility of iPSC models in modeling cellular mechanisms of therapeutic compounds (Marchetto et al., 2017).

Additionally, there is evidence of increased activation of microglia and astrocytes in the brain, suggesting an inflammatory response. Co culture models investigating the interactions between neurons and astrocytes from individuals with idiopathic ASD using iPSCs have also been illustrative. ASD-derived neurons showed a significant decrease in synaptic gene expression and protein levels, glutamate neurotransmitter release, and reduced spontaneous firing rate. ASD-derived astrocytes interfered with proper neuronal development, but this was rescued with control-derived astrocytes. Finally, blocking interleukin-6 secretion from astrocytes resulted in increased synaptogenesis in ASD-derived co-cultures (Russo et al., 2018).

In ASD, one of the key cellular phenotypes is altered synaptic formation and pruning. This leads to changes in neuronal connectivity, which are thought to underpin many of the behavioral characteristics of ASD (Beopoulos et al., 2022). A 2021 study exploring the role of the innate immune complement system in ASD found that complement c4 was reduced in iPSC-derived astrocytes from people with ASD. Since astrocytes participate in synapse elimination, diminished c4 levels are associated with defective synaptic pruning, thus leading to atypically enhanced brain connectivity in ASD (Mansur et al., 2021).

Griesi-Oliveira et al. (2015) performed the inaugural iPSC research on non-syndromic ASD, targeting a unique TRPC6 mutation. They discovered that disruption in TRPC6 caused diminished calcium signaling and led to abnormal neuronal development, marked by reduced neurite length and complexity, along with a scarcity of dendritic spines. Correcting these defects was achievable through either activating TRPC6 or enhancing its expression, highlighting TRPC6’s significance in ASD’s neurodevelopmental mechanisms.

Emerging research into ASD points to neuroinflammation and immune system dysregulation as pivotal factors in understanding the disorder’s causes and progression. Research utilizing iPSC-derived neurons highlights the critical role of interferon-𝛾 (IFN-𝛾) signaling in neurodevelopmental disorders. IFN-𝛾 influences neurite outgrowth by up-regulating MHCI genes via mediation by promyelocytic leukemia protein nuclear bodies. Additionally, it alters gene expression associated with ASD and SCZ, underscoring the interaction between genetic and environmental factors (Warre-Cornish et al., 2020).

Imbalances in the excitatory-inhibitory neurotransmitter systems, particularly involving glutamatergic and GABAergic neurons, are also commonly observed in ASD. These imbalances can affect neural network functioning and are linked to the sensory and behavioral symptoms characteristic of the disorder. In a study utilizing co-cultures of iPSC-derived glutamatergic and GABAergic neurons, the focus was on Cadherin-13’s (CDH13) pivotal role in regulating the excitation/inhibition (E/I) balance. Findings indicate that a lack of CDH13 in GABAergic neurons leads to a disrupted E/I balance, primarily through enhanced inhibition (Wang et al., 2023).

An examination of the transcriptome analysis was performed between normal controls and iPSC-derived neuronal cells from normocephalic ASD. Researchers identified dysregulation in gene modules associated with protein synthesis in neuronal progenitor cells (NPC), synapse/neurotransmission, and translation in neurons. Proteomic analysis of NPCs showed potential molecular links between these dysregulated modules in NPCs and neurons. A synapse-related module was consistently upregulated in iPSC-derived neurons (similar to fetal brain expression) and downregulated in postmortem brain tissues of ASD patients, pointing to its potential as an ASD biomarker and therapeutic target due to its varying dysregulation across developmental stages in ASD individuals. This research demonstrates that traditional 2D models, when integrated with newer technologies such as RNA-seq, transcriptomic analysis, differential gene expression profiling, and the prediction of temporal and regional identity of iPSC-derived neurons through machine learning algorithms, can still provide valuable insights into complex biological processes (Griesi-Oliveira et al., 2021).

### Schizophrenia

SCZ is characterized by significant disruptions in neuro-transmitter systems, particularly the overactivity of dopamine pathways in the brain. This over activity, especially in regions associated with reward and motivation, is believed to contribute to symptoms like hallucinations and delusions. Structurally, SCZ is often associated with reductions in the volume of the cerebral cortex and alterations in the structure of specific brain regions, including the thalamus and hippocampus (Nakamura and Takata, 2023). These changes may underlie many of the cognitive impairments observed in SCZ. iPSC models of SCZ have revealed alterations in neurogenesis, neuronal maturation, neuronal connectivity, synaptic impairment, mitochondrial dysfunction, glial cell dysfunction, and developmental impairments mediated by miRNAs (Dubonyte et al., 2022).

Aberrant activation of inflammasomes, dysregulation of glial cells, and brain inflammation have been implicated in the pathophysiology of SCZ (Akkouh et al., 2020). Astroglia and microglia, as the brain’s resident immunocompetent cells, are central to ongoing research into immune abnormalities associated with SCZ. Notably, iPSC-derived astrocyte and astroglia studies (Akkouh et al., 2020; Akkouh et al., 2021; Szabo et al., 2021) highlight the critical involvement of these cell types in SCZ’s underlying mechanisms. This research highlights the importance of glial cells in mediating the inflammatory responses that may contribute to the disorder’s development and progression. Using SCZ iPSCs differentiated into astrocytes, researchers identified genes that were differentially expressed during development in SCZ-astrocytes compared to controls. Then, they confirmed that these genes were up-regulated in the medial prefrontal cortex, striatum, and temporal lobes which have been previously implicated in SCZ. Additionally, SCZ astrocytes showed altered calcium signaling, decreased glutamate uptake, and metalloproteinase activity (Szabo et al., 2021). Research has also shown that inflammatory modulation, specifically through IL-1B exposure, affects iPSC-derived astrocytes differently in SCZ patients compared to healthy controls (Akkouh et al., 2020). IL-1B exposure altered pathways related to innate immune responses, cell cycle regulation, and metabolism in both SCZ and control astrocytes. However, significant differences were found in the expression of HILPDA and CCL20 genes which exhibited reduced up-regulation after IL-1B treatment in SCZ astrocytes compared to healthy controls. This implies that the dysregulated immune activation and inflammation in SCZ could be, in part, related to a possible disruption in the astroglia-CCL20-CCR6-Treg axis (Akkouh et al., 2020).

Another study conducted a comparative analysis of miRNA expression in iPSC-derived astrocytes from SCZ patients and healthy controls, both at baseline and after inflammatory stimulation. They identified several miRNAs that exhibited significantly lower baseline expression relative to controls (Akkouh et al., 2021). These findings were validated by analyzing gene expression in blood samples from a large group of SCZ patients and controls. After screening for the expression of specific gene targets of the miRNAs, they found several genes that were down regulated in the blood of SCZ patients compared to controls. These findings highlight the critical role of astrocytes and astroglia in mediating synaptic and inflammatory processes in SCZ, presenting new avenues for understanding and potentially treating the complex pathobiology of this disorder (Akkouh et al., 2020; Akkouh et al., 2021; Szabo et al., 2021). Studies involving cultured interneurons differentiated from iPSCs of SCZ patients both showed mitochondrial dysfunction, altered synaptic structure, and oxidative stress. Additionally, both these studies showed that these neuronal deficits could be rescued with therapeutics which target oxidative stress or mitochondrial dysfunction (Kathuria et al., 2019; Park et al., 2020).

Cortical interneurons (cINs) derived from patient iPSCs exhibited lower levels of the enzyme glutamate decarboxylase 67 and synaptic proteins, including gephyrin and neuroligin-2 (NLGN2). When these interneurons were co-cultured with excitatory cortical pyramidal neurons also derived from SCZ patient iPSCs, a notable reduction in synaptic puncta density and a decrease in action potential frequency was observed. Crucially, the reduction in NLGN2 was identified as a key factor in diminishing puncta density, as its overexpression successfully reversed these deficits. Furthermore, treatment with the antioxidant *N*-acetylcysteine not only increased NLGN2 expression levels but also improved synaptic deficits and signs of oxidative stress (Kathuria et al., 2019). In a related study, cortical cINs derived from iPSCs from both healthy controls and individuals with SCZ were co-cultured with activated microglia to examine the effects of prenatal immune activation, a known SCZ risk factor. The interaction with activated microglia led to disrupted metabolic pathways, impaired mitochondrial function, reduced arborization, and decreased synapse formation and synaptic GABA release in cINs. Mitochondrial dysfunction and reduced branching in neurons, induced by activated microglia, could be reversed with alpha lipoic acid and acetyl-L-carnitine. While healthy control neurons were affected by the activated microglia, neurons from individuals with SCZ were not, suggesting a unique interaction between SCZ genetics and environmental factors. Even after removing the microglia, SCZ neurons exhibited lasting metabolic issues, demonstrating a potential vulnerability specific to SCZ (Park et al., 2020). These studies identify synaptic and mitochondrial dysfunction in cortical interneurons as key mechanisms in SCZ, suggesting potential avenues for future therapeutic intervention (Kathuria et al., 2023).

**Figure 1:**
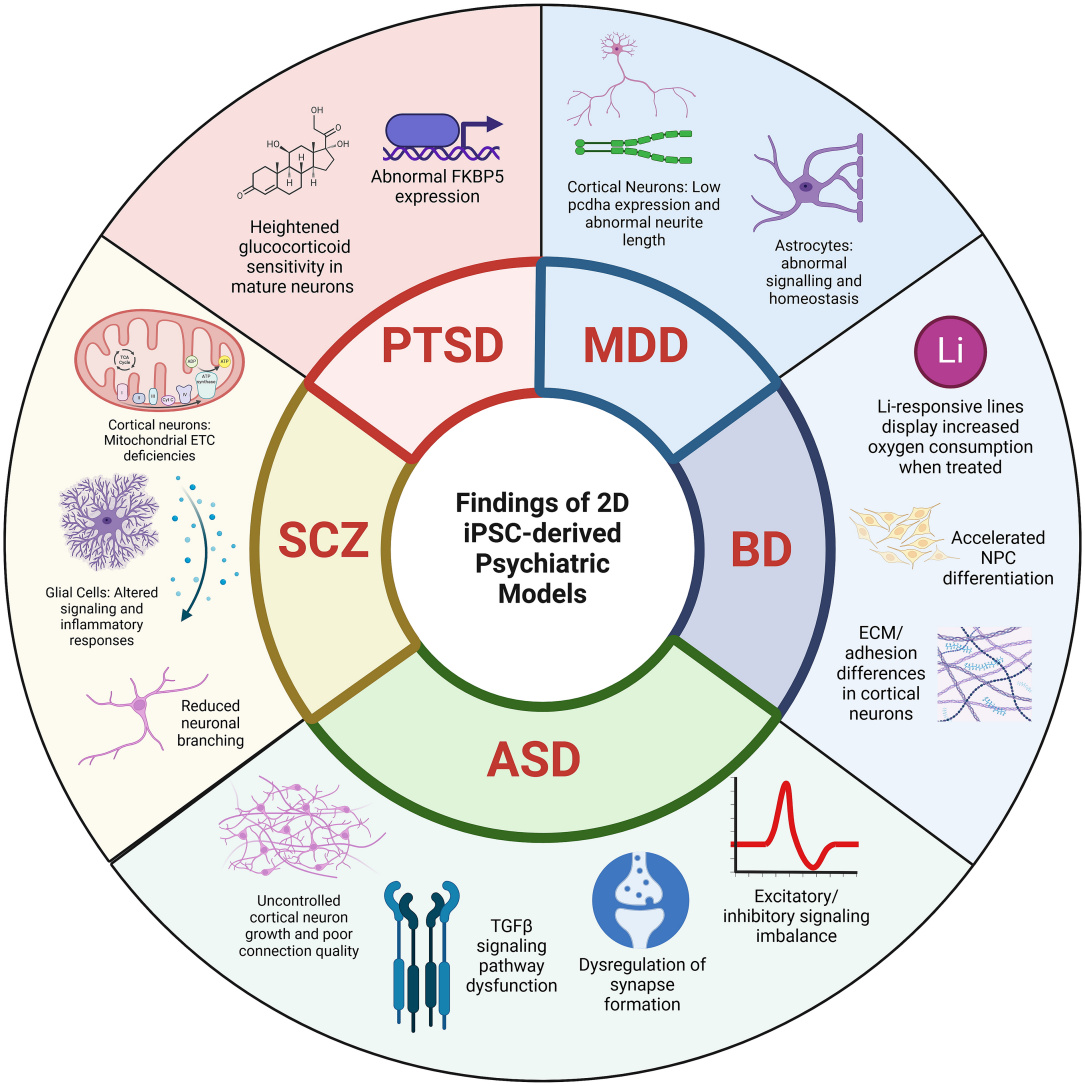
Key cellular and molecular findings from 2D-induced pluripotent stem cell (iPSC)-derived models of psychiatric disorders share critical abnormality features in neuronal function, signaling pathways, and cellular phenotypes among diverse disorders: PTSD (post-traumatic stress disorder), MDD (major depressive disorder), BD (bipolar disorder), ASD (autism spectrum disorder), and SCZ (schizophrenia).

Finally, recent studies showed that SCZ-related deficits in dendritic spine density are associated with altered gene expression, particularly the NRXN3 204 isoform, in iPSC-derived cortical neurons (Figure 1). The findings align with postmortem analyses and suggest that modulating NRXN3 204 expression, potentially with treatments like clozapine, could address synaptic abnormalities in SCZ. Another critical aspect of SCZ is the dysregulation of glutamate neurotransmission. This involves particularly the NMDA receptors, which play a significant role in synaptic plasticity and memory.

### Bipolar disorder

BD presents a complex picture involving neurotransmitter imbalances and neural plasticity dysfunctions. Neurotransmitters like serotonin, dopamine, and norepinephrine play a crucial role, similar to their involvement in anxiety disorders (ADs). Fluctuations in these neurotransmitter levels are thought to contribute to the mood swings experienced in BD. Additionally, abnormalities in neural plasticity are a key feature, often indicated by altered levels of neurotrophic factors such as BDNF (Brain-Derived Neurotrophic Factor). These changes can affect neuronal growth and survival, impacting mood regulation and cognitive functions (Scaini et al., 2020).

Dysfunctions in ion channels, particularly those involved in calcium signaling, are also linked to BD, influencing neuronal excitability and neurotransmitter release. Store-operated calcium entry (SOCE) is a mechanism for regulating intracellular calcium levels and is crucial for central nervous system development and many aspects of neuroplasticity (Rosenberg and Spitzer, 2011). Calcium and developmental dysregulations related to SOCE were noted in BD-NPC and cortical-like glutamatergic neurons derived from BD-iPSC’s. Additionally, RNA-sequencing identified a unique transcriptome profile in BD-NPCs, suggesting that BD resulted in accelerated neurodifferentiation. The researchers also identified high expression of microRNAs let-7 and miR-34a in BD NPCs and BD neurons, respectively. Both have been implicated in BD etiology and neurodevelopmental deviations (Hewitt et al., 2023).

Similar to SCZ, astrocytes and their impact on the inflammatory state of the brain have been implicated in BD. BD-iPSC derived astrocytes exhibited altered transcriptional activity and were less supportive of neuronal activity both at baseline and with inflammatory stimulation compared to controls. Additionally, neuronal activity decreased during co-culture with IL-1 𝛽-stimulated astrocytes Administration of an IL-6 blocking antibodies rescued this neuronal activity, suggesting that IL-6 partially mediated the effects of activated astrocytes on neuronal activity (Vadodaria et al., 2021).

iPSCs derived from patients with BD have been utilized to better understand the cellular behaviors and differential gene expression between lithium responders (LR) and non-responders (NR) (Stern et al., 2018; Niemsiri et al., 2023; Osete et al., 2021). This research has generated novel insights into the molecular mechanisms of mood stabilizer function with focal adhesion, oxidative phosphorylation, and spliceosome modifications being implicated in therapeutic response.

In 2023, researchers used a network-based multi-omics approach to analyze iPSC-derived neurons from LR and NR which revealed significant differential gene expression related to focal adhesion and ECM. They posit that disrupted focal adhesions could impact axon guidance and neuronal circuits which may underpin mechanisms of response to lithium and underlying BD (Niemsiri et al., 2023).

BD-iPSC derived hippocampal dentate gyrus neurons have been shown to be electrophysiologically hyperexcitable. Functional analysis showed that there are significant differences in intrinsic cell parameters between LR and NR patients, suggesting the existence of BD subtypes. One discovery across both groups is the presence of a large, fast after-hyperpolarization, underscoring its potential role in BD’s neurobiology. Furthermore, lithium’s ability to modulate this hyperexcitability in LR patients reaffirms its therapeutic relevance, offering insights into BD’s underlying mechanisms and treatment responsiveness (Stern et al., 2018).

### Anxiety disorders

ADs are characterized by a complex interplay of neurotransmitter imbalances and structural brain alterations. From a structural perspective, ADs are frequently associated with changes in the hippocampus and amygdala, key regions for processing fear and stress. The hippocampus, vital for memory and emotional regulation, may undergo functional and structural modifications, while the amygdala, central to the fear response, can exhibit hyperactivity. Chronic stress, a common trigger for ADs, can lead to the dysregulation of the hypothalamic–pituitary–adrenal axis, influencing cortisol levels and further contributing to the symptomatology of these disorders (Ghasemi et al., 2022).

Neurotransmitters such as serotonin, GABA (gamma-aminobutyric acid), and norepinephrine are central to the development of these disorders. Serotonin, often linked to feelings of well-being, is typically found to be dysregulated in anxiety conditions. GABA, the brain’s primary inhibitory neurotransmitter, plays a crucial role in managing neuronal excitability and, when imbalanced, can lead to heightened anxiety states. Norepinephrine, which is involved in the body’s stress response, is also often dysregulated (Ghasemi et al., 2022).

Animal models of anxiety, compared to all the other mental disorders, are generally the most amenable to translation because fear and anxiety are highly tractable in the laboratory (Grillon et al., 2019). However, the differences between human and animal biology has limited their effectiveness in identifying psychopharmacological treatments and limited the ability to test more novel treatments like neurostimulation. Furthermore, non-human animal models fall short due to the dubious nature of ascribing human psychiatric diagnosis criteria to a non-human animal and the inability to observe complex cognitive processes in animal test subjects that cannot self-report their mental state.

### Depression

In depression, a notable reduction in hippocampal volume is a common observation. This reduction is critical, as the hippocampus plays a significant role in emotional processing and memory formation. The prefrontal cortex, integral for mood regulation and decision-making, also shows altered activity and, in some cases, atrophy in individuals with depression. These structural changes are thought to contribute to the cognitive and emotional symptoms characteristic of depression (Fox and Lobo, 2019).

Neurotransmitter systems involving serotonin, norepinephrine, and dopamine are frequently implicated in depression. Imbalances in these neurotransmitters can disrupt mood regulation and lead to depressive symptoms. Moreover, depression is often associated with a reduction in neuroplasticity. This includes decreased neurogenesis, particularly in the hippocampus, and a reduction in synaptic plasticity, which can affect learning, memory, and mood regulation (Fox and Lobo, 2019).

While iPSC models for depression are less commonly employed than for BD, ASD, or SCZ, their application in exploring the differential effects of therapeutics for MDD has already proven valuable. For instance, prefrontal cortical neurons from depression patient iPSC’s were cultured and subsequently used to screen for the effectiveness of bupropion, a commonly used antidepressant. Biomarkers specific for response to the medication were discovered including specific gene expression alterations, synaptic connectivity and morphology changes (Avior et al., 2021). In another study, serotonergic neurons from MDD iPSCs in both SSRI-remitters and non-remitters were generated. Then, their resistance to selective serotonin re-uptake inhibitors (SSRIs) was studied. Non-remitters were found to have altered neurite growth and morphology downstream of lowered expression of protocadherin alpha genes compared to controls and remitters. Knockdown of protocadherin alpha genes improved iPSC-derived neurite length and morphology further implicating its role in SSRI resistance (Vadodaria et al., 2019). A related study using iPSC derived neurons from MDD SSRI NRs displayed serotonin-induced hyperactivity downstream of up-regulated serotonergic receptors compared to SSRI-Rs (Vadodaria et al., 2019). This result implies that differences in serotonergic neuron morphology and the resulting circuitry may impact SSRI resistance in MDD patients (Vadodaria et al., 2019).

These studies show how iPSC depression models can be utilized to predict patient response to psychiatric medications and probe MDD’s molecular phenotype. Another related avenue of research is the relationship between chronic stress and MDD. To model this interaction, iPSC-derived astrocytes were treated with cortisol for 7 days to mimic chronic stress exposure *in vitro*. Then, differentially expressed genes (DEGs) between MDD-iPSCs astrocytes and control astrocytes in response to stress were identified. The researchers found that genes related to GPCR ligand binding, synaptic signaling, and ion homeostasis were differentially expressed. This modeling approach, alongside the pinpointed DEGs, warrant future study to further elucidate the cellular and molecular mechanisms that mediate the impact of stress in the brain (Heard et al., 2021). Similar research looking at the importance of glucocorticoid signaling in iPSC-derived cortical neurons identified the gene CDH3 as being critically implicated in the cellular response to chronic stress (Chatzinakos et al., 2023). Overall, iPSC models hold potential in unraveling the mechanisms behind MDD, offering insights into antidepressants effectiveness, SSRI resistance, and the relationship between chronic stress and MDD.

### Post-traumatic stress disorder

PTSD shares some cellular phenotypes with depression, such as a reduced volume of the hippocampus. This reduction may contribute to the memory disturbances typically observed in PTSD. The amygdala, crucial for processing fear and emotional responses, often exhibits heightened activity in PTSD, correlating with the heightened vigilance and fear responses characteristic of this disorder. Another key feature of PTSD is the dysregulation of the HPA axis, which leads to abnormal cortisol patterns (Bailey et al., 2013). Glucocorticoid dysregulation can affect the body’s stress response system, contributing to the persistence of PTSD symptoms and impacting other bodily systems. To simulate glucocorticoid dysregulation seen in PTSD, iPSCs were differentiated into cortical neurons under dexamethasone (DEX) treatment, activating glucocorticoid receptors to mimic stress responses. This method also involved tracking gene expression changes through the stages of differentiation from iPSCs into neural stem cells, immature neurons, and finally cortically neurons. The analysis identified DEGs among the DEX treated neurons including canonical glucocorticoid receptors genes like FKBP5 and non-glucocorticoid genes like the serotonergic gene TPH2. Additionally, the expression of glucocorticoid-related genes, crucial for understanding PTSD, showed greater sensitivity in mature neural cells compared to their immature counterparts. This suggests that mature neural cells provide a more effective model for studying PTSD *in vitro* (Pernia et al., 2021). In 2022, a study demonstrated that glutaminergic neurons derived from iPSC lines from PTSD patients exhibit a heightened sensitivity to levels of glucocorticoids in the body, especially at low levels (Seah et al., 2022). A similar study found that glucocorticoid signaling pathways were differentially enriched in PTSD in iPSC-derived cortical neurons compared to MDD, especially in excitatory neurons (Chatzinakos et al., 2023). As the majority of trauma survivors do not develop clinically diagnosed PTSD, these findings may support the diathesis-stress model of many psychiatric disorders in which certain individuals are born with cellular phenotypes that predispose them to developing a disorder when exposed to a certain environmental trigger.

### Limitations of modeling psychiatric disorders in 2D

While studies of 2D *in vitro* iPSC models of psychiatric disorders have yielded valuable findings, they face the fundamental limitation of failing to capture the full complexity of cell–cell interactions that exist in 3D, *in vivo* settings. In 2D culture, neurons can only form connections in 2D planar space, due to being restricted by the surface of the culture plate below them. The removal of the third dimension not only greatly reduces the number of possible connections between the cultured neurons, but also causes every cell to be in contact with an unnatural, stiff, and flat surface. Most psychiatric disorders display some degree of alterations to cell phenotype in the form of abnormal growth and development of neuronal connections and abnormal neurotransmitter signaling, and 2D conditions cannot fully capture these complexities due to the aforementioned restrictions on growth and cell–cell communication.

## 3D models: brain organoids

Organoids are 3D structures derived from hPSCs in vitro that mimic the complex biological functions of real organs. This technology represents a significant leap from traditional two-dimensional cell cultures, offering a more accurate and dynamic model for studying patient-specific developments, disease processes, and drug responses. Typical brain organoid models are differentiated from iPSCs in a stepwise manner and grown in Matrigel or other ECM analog using Lancaster’s protocol and the hanging drop method.

In this review, we focus on psychiatric disorders and the advances in integrating cerebral organoids into the disease-modeling research framework. We discuss the typical cellular phenotypes of multiple psychiatric disorders such as ASD, SCZ and BD (Table 2). We aim to identify the advantages of 3D modeling over 2D modeling and discuss the recent studies performed using these models in various neuropsychiatric diseases. Psychiatric disorders are as such extremely difficult to study and its diagnosis involves components like environment, mood and psychosis. It is very difficult to translate this in an accurate manner to gain complete understanding. The absence of markers like cerebrospinal fluid, brain imaging and EEGs unlike those in neurological disease complicates it further. One way to robustly replicate the disorder is through using 3D models like cerebral organoids as they have the ability to self-organize. These models focus on modeling cellular aspects of the disorder such as cell proliferation, migration, lineage trajectory and differentiation.

**Table 2:** Summary of cellular phenotypes in 3D models.

Disease	Cell type	Region	Observations/Findings	References
BD	Neural Progenitor Cells	Cortex	Store-operated Ca release dysregulated and attenuated in BD NPC lines compared to controls. Thinning of subventricular zone observed in BD organoid lines.	Hewitt et al. (2023)
BD	Neurons	Cortex	Enrichment of genes related to ion storage and homeostasis in BD-derived cortical organoids. Store-operated Ca release dysregulated and attenuated in BD cerebral organoids. BD-derived cortical organoids contain lower proportions of neurons and elevated numbers of radial glial cells. Decreased neuron excitability observed in BD-derived cortex organoids but excitability was rescued by treatment with lithium. DEGs between treated and untreated organoids associated with Na + homeostasis and regulation of IL-𝛽 and TNF-𝛼.	Hewitt et al. (2023), Osete et al. (2023), and Jourdon et al. (2023)
BD	Excitatory Cortical Neurons	Cortex	BD Type I-derived organoids display downregulation of cell adhesion associated with abnormal NCAN expression, and downregulation of genes associated with GABA uptake/release. ER-Mitochondria contact sites markedly reduced in BD-derived organoids compared to controls, in both perinuclear region and neurites.	Kathuria et al. (2020)
SCZ	Neurons	Cortex (Dorsal Forebrain), Ventricles	GWAS studies by Kathuria et al. (2020) reveal 23% of genes are differentially expressed in SCZ organoids compared to controls. Significant decreases in mitochondrial activity and cellular respiration are observed in SCZ organoid lines. MEA studies reveal diminished response to stimulation/depolarization in SCZ organoids. BRN2 and PTN are depleted in Scz neurons relative to controls.	Notaras et al. (2022) and Kathuria et al. (2020)
SCZ	Non-Neurons	Ventricles	Elevated numbers of cells in ventricular regions of SCZ organoids directed toward myeloid, mural, and endothelial lineages in comparison to controls.	Notaras et al. (2022)
SCZ	Neurons	Thalamus, Cerebellum	Impaired connection between medial dorsal nucleus of the thalamus and cerebellum	Urenda et al. (2023)
ASD	Excitatory Neurons	Cortical Plate	Elevated numbers of cortical plate excitatory neurons (ENs) in macrocephalic ASD lines at the expense of preplate ENs, which were diminished. Cortical interneurons are also overproduced in comparison with controls due to DLX6 overexpression.	Quadrato and Birtele (2023) and Huang et al. (2022)
ASD	Radial Glial Cells	Cortical Plate	Lamination of cortical plate is reduced, and cortical projection neuron maturation is accelerated in SYNGAP1 haploinsufficient organoids. Similar findings observed in vivo in mice.	Quadrato and Birtele (2023)
ASD	NPCs	General	Excessive growth of NPCs due to mutations in genes such as SUV420H1, CHD8, and PTEN associated with ASD risk and macrocephaly.	Paulsen et al. (2020), Rabeling and Goolam (2023), and Paulsen et al. (2022)
ASD	GABAergic Neurons	Cortex	Overexpression of GABAergic pathways in ASD organoids results in heightened percentages of GABAergic neurons (as high as 50% of all cells in the organoid) compared to controls.	Paulsen et al. (2020) and Paulsen et al. (2022)

Specifically, cerebral organoids have been used by researchers to understand and observe abnormalities in abnormal psychiatric conditions, model brain development, and as frameworks for preclinical drug testing regimens and personalized medicine (Sun et al., 2021). Since commonly highlighted psychiatric disorders such as ASD, SCZ, AD, depression, PTSD and BD involve multiple brain regions, cerebral organoids specific to particular regions can be cultured using human iPSCs and fused to form assembloids. This is a powerful tool for scientists to identify multiregional affects and how the connectivity between them contributes toward the disorder, defects in cellular organization and the underlying biological (Zhang et al., 2021; Urenda et al., 2023).

### Modeling autism spectrum disorder using organoids

ASD are characterized by an array of symptoms that can range from mild to severe, including repetitive behaviors, social interaction difficulties, and impaired neurodevelopment. This review focuses on idiopathic autism spectrum disorders, which occur spontaneously and have unclear and polygenic causal factors, intriguing scientists. Studying these complexities requires a comprehensive understanding of brain development and the relationship between cellular and molecular results associated with clinical symptoms.

Previous sections have highlighted extensive studies using 2D and animal models, but the limitations of both hinder the clinical translation of findings. Studies have shown that culturing cells in monolayers alters their genetic expressions. In rodent models of ASD, neurons originate from the subventricular zone, whereas in humans, they originate from proliferative regions outside this zone (Wang et al., 2011). To bridge species-specific differences, human brain organoids derived from iPSCs are now being modeled to gain deeper insights into the etiology of autism spectrum disorder.

Macrocephaly is one of the most well-studied phenotypes associated with ASD, leading researchers to use organoids in studying its etiology. Brain organoids derived from individuals with macrocephalic ASD show larger sizes compared to control organoids due to the involvement of tumor suppressors such as PTEN, CHD8, and RAB39B. This increase in organoid size is also attributed to the overexpression of neural progenitor cells (NPCs) caused by impaired maturation during development (Paulsen et al., 2020). In particular, organoids derived from a cell with shortened form of CHD8 contribute to the increase in organoid size and are implicated in macrocephalic ASD, along with abnormal PTEN and SUV420H1 expression, an imbalance of neuronal types and increased differentiation of GABAergic neurons (Rabeling and Goolam, 2023; Paulsen et al., 2020; Paulsen et al., 2022).

Additionally, organoid models have implicated imbalances in neuronal subtypes as possible causes for behavioral symptoms of ASD. The overexpression of upper-layer colossal projection neurons, which are responsible for human social behavior, is observed in ASD and can be further explored using organoid models. Cortical organoid models were created to illustrate that the expression of SYNGAP1 in human radial glia is reduced in individuals with ASD. This reduction impacts the lamination of the cortical plate and influences the maturation of cortical projection neurons (Quadrato and Birtele, 2023). Additionally, increased expression of DLX6 leads to the overproduction of cortical interneurons compared to control organoids. Cortical organoids derived from iPSCs affected by idiopathic ASD show upregulated expression of the transcription factor forkhead box G1, leading to shortened cell cycle length, abnormal cell proliferation, and unbalanced neuron differentiation. The imbalance between inhibitory and excitatory neurons during early development suggests accelerated brain growth in the second trimester (Ilieva et al., 2018). This imbalance is also studied in forebrain organoids, comparing the cellular and molecular profiles of macrocephalic and non-macrocephalic ASD. The analysis concludes that there is an overlap between the two in terms of upregulated genes in transcription, with patient-derived organoids showing decreased cortical area-specific transcripts (Jourdon et al., 2023). The size of organoids is also assessed using proteomic techniques and time-point assessments (Ilieva et al., 2022).

### Understanding schizophrenia through organoid modeling

SCZ, a neurodevelopmental disorder with an varied molecular origin, was initially modeled in 2011 using patient-derived hiPSCs carrying mutated DISC1 genes. Subsequent research delved into investigating dysregulation in synapse-associated gene transcription, defective synaptic vesicle release, cell cycle regulation, and its impact on cortical size. Progress in this area led to the utilization of 3D modeling techniques, specifically cerebral organoids, to dissect the cellular phenotype of SCZ. In 2011, the Gage lab demonstrated neuronal phenotypic changes derived from patient cells, revealing reduced neuronal connectivity, decreased levels of PSD-95 protein, alterations in glutamate expression, and dysregulation in cAMP and Wnt signaling pathways. These advancements transitioned into using organoid models and conducting cell patterning assays, which identified decreased connectivity between the medial dorsal nucleus of the thalamus and cerebellum in schizophrenic patients (Urenda et al., 2023). Presently, organoid models mimicking neuropathology of SCZ are employed to assess risk factors such as amalgamate, PTN, and BRN2 (Notaras et al., 2022). Other research has focused on patterning, time-dependent organoid generation, abnormal distribution of proliferating (Ki67+)neural progenitor cells, and the role of genes like FGFR1 in cortical development (Stachowiak et al., 2017). In addition, studies have identified DEGs associated with significant decreases in mitochondrial activity and cellular respiration in SCZ organoid lines (Kathuria et al., 2020; Kathuria et al., 2023).

### Organoids model for bipolar disorder

BD, a neuropsychiatric condition, remains poorly understood in terms of its structural, cellular, and circuit-level characteristics. The absence of clear diagnostic biomarkers and the complexity of its behavioral traits pose significant challenges for treatment (Quadrato et al., 2016). Given its involvement in higher-order brain functions and the presence of multiple cell types across various brain regions like the thalamus, cortex, and basal ganglia, utilizing 3D models such as cerebral organoids offers a potent strategy for analysis. Studies on patient-derived samples have revealed dysregulated genes and signaling pathways such as the Wnt pathway, which has also been observed in other neuropsychiatric disorders. BD-derived organoids exhibit reduced neuron count, resulting in decreased excitability and network activity, along with smaller size. Additionally, there is evidence of overexpression of genes related to membrane receptors and calcium channels (Villanueva, 2023). Lithium has been used as a treatment in BD for its mood-stabilizing properties; however, its mechanism was not completely understood until studies were performed using brain organoids. In patient derived organoids, the administration of lithium increases excitability, neuroprotection, mitochondrial reverse capacity and regulated pro-inflammatory cytokine secretions. This helped to identify Li-associated DEGs (differentially expressed genes) which can serve as targets for drug delivery (Osete et al., 2023). iPSC-derived cerebral organoids from patients serve as a valuable model to understand aberrations in ER biology and electrophysiological responses, as well as the dysregulation of cell adhesion, upregulation of immune signaling genes, and neurodevelopmental aspects in BD (Whiteley et al., 2022; Kathuria et al., 2020).

### Limitations of 3D models

Despite their numerous advantages over 2D models, 3D models of psychiatric diseases face a number of limitations that must be addressed before they can become more widely used in basic and clinical research. The small size of organoids means that there is still a significant discrepancy between the behavior of organoid cultures and the complex electrophysiology of the human brain. To addressing this limitation, scientists are now using standardized kits to perform long term culturing. This provides a platform for organoids to grow in size and enhance neural complexity to mimic the human brain. Notably, the existing body of research concerning cerebral organoids and their applications has focused primarily on cortex neurons. As dysfunctional circuits involving multiple non-cortical brain regions have been theorized to contribute to the etiology of many psychiatric disorders, we speculate that the use of assembloids of both cortical and non-cortical brain regions would improve the similarities to physiological conditions. Furthermore, organoid models often display a greater degree of structural similarities to fetal organs rather than their adult counterparts, as a consequence of being grown from naive iPSC cultures over the span of only a few months. Specifically, this can be problematic for the prospects of using organoid models in psychiatric research, as most mental disorders have an onset between adolescence and young adulthood. Now, researchers are using iPSCs derived from older patients to differentiate them into organoid models.

Finally, because the body of research using 3D models is relatively small compared to that concerning 2D iPSC models, there exist fewer methods for characterization and analysis. For example, 2D iPSC neuron cultures are commonly analyzed using microelectrode arrays (MEAs). These systems which are designed for recording electrical potentials of flat monolayer cultures are not suitable for doing the same with 3D organoids. However, some recent research such as that of Huang et al. (2022). Salick et al. (2021) has explored multistep microfabrication methods for microscale Shell MEA devices that can wrap around a single organoid, transmitting measurements from multiple spatial directions in a limited capacity. The group demonstrated that this device could be used to measure the signals from a single cerebral organoid, both before and after treatment with glutamate.

## Future of 3D models

### Drug testing and personalized medicine

In the academic realm, brain organoids are employed to investigate the genotype and phenotype of complex neurodegenerative and neuropsychiatric conditions. By comparing control organoids with disease organoids, researchers aim to identify new drug targets for these disorders. Utilizing 3D models enables the examination of drug components, with the goal of potentially replacing the need for animal models in drug testing.

For instance, the effects of lithium on mechanisms and pathways implicated in BD have been explored using patient-derived brain organoids. Similarly, CRISPR technology is utilized to suppress entry receptors of the Zika virus in cerebral organoids. Neurotherapeutic companies utilize iPSC-derived cerebral organoids for phenotypic-based drug discovery targeting psychiatric diseases. Collaborations between institutes like IMBA and academia fund studies on loss-of-function mutations in SCN1A, a sodium channel gene associated with epilepsy in Dravet syndrome (Yang et al., 2023).

The complex nature of idiopathic autism spectrum disorder presents challenges in using patient-derived organoids for drug testing. However, for syndromic ASDs, cerebral organoids serve as useful platforms. Drug testing on organoids has shown promising results, such as the reduction in tumor size with mTOSR complex inhibitor and EGFR kinase inhibitors, Everolimus and Afatinib, respectively. Additionally, experiments administering topoisomerase inhibitors to cerebral organoids have revealed reduced expression of UBE3A-ATS, indicating potential implications for autism spectrum pathology. Investigations into IGF treatments for ASD are also underway using these models (Rabeling and Goolam, 2023).

Transcriptomic profiling of iPSC-derived telencephalic organoids from pediatric BD patients has identified dysregulated PLXNB1 gene signaling as a contributing factor (Shen et al., 2024). Furthermore, leaky channels implicated in BD have been studied using patient-derived models (Yuan et al., 2023). Wolfram syndrome is a neuropsychiatric disorder caused due to WFS1 deficiency in astrocytes, this causes delayed neuronal differentiation, disrupted synapse formation, hampers neurite growth. Cerebral organoids from hESC patient cells are used to study Riluzole, a drug which induces EAAT2 restoration in astrocytes and reverse disorder effect (Chen et al., 2019). However, further examination is required to fully assess the utility of these models in drug testing and discovery.

Brain organoids derived from patient-specific iPSCs offer a valuable platform for personalized medicine. As previously mentioned, they represent a potent tool for drug development targeting conditions like Zika virus-associated microcephaly, ASD, and glioblastoma. Glioblastoma, an unresectable brain cancer characterized by high genetic diversity, can be better understood by isolating glioma stem cells from patients and cultivating glioma organoids. These organoids mimic key features of gliomas *in vivo*, including cell types and hypoxic gradients (Zhou et al., 2021). Additionally, the introduction of the HRas oncogene through CRISPR/Cas9-mediated homogenous recombination has been utilized to generate glioma organoids. These models have been instrumental in studying factors influencing the tumor microenvironment (Zhou et al., 2021). Besides brain organoids, 3D models derived from other tissues like the colon, liver, and prostate have also demonstrated promising applications in personalized medicine (Sahu and Sharan, 2020). Establishing biobanks of these organoids is crucial for enabling high-throughput screening, epigenomic analysis, and transcriptional profiling. However, the creation of biobanks specifically for patient-derived organoids remains a pending task (Chhibber et al., 2020).

### Neural toxicology

The resemblance of 3D brain organoids to human brain architecture positions them as valuable tools in drug development, particularly for evaluating neurotoxicity and mimicking pathophysiology. Organoids circumvent ethical limitations on the use of human embryos, enabling direct administration of chemicals or drugs to assess their effects on function, morphology, and transcription. For instance, drugs prescribed during pregnancy can influence fetal neural development, increasing the risk of neurological disorders (Fan et al., 2022; Liu et al., 2019).

Several studies have utilized brain organoids to investigate neurotoxicity. In 2019, vincristine exposure was found to inhibit tubulin and fibronectin development in cerebral organoids, demonstrating dose-dependent neurotoxic effects (Bu et al., 2020). A 2020 study on acrylamide, a common food contaminant, revealed prolonged exposure induced tau hyperphosphorylation, reduced neuronal differentiation, and increased NRF2 activity (Cui et al., 2021). Comparative studies in 2021 showed that 4-hydroxybenzophenone exposure reduced proliferation and caused necrosis in organoids, while postnatal exposure in rodents led to apoptosis in hippocampal neural stem cells and cognitive deficits (Arzua et al., 2020).

Organoids have also been pivotal in modeling fetal alcohol spectrum disorders (FASD), with patient-derived organoids revealing alcohol-induced mitochondrial dysfunction, metabolic stress, along with alterations in the expression of 199 genes out of 17,195 involved in neurodevelopment (Mendez et al., 2023). Similarly, prolonged exposure to cadmium, an environmental pollutant, led to reduced organoid size, impaired GFAP and CTIP2 expression, altered SOX2 neural progenitor production, and disrupted synaptogenesis (Parmentier et al., 2023). Moreover, effects of morphine on early development of the brain is being studied using organoids (Huang et al., 2024). Multielectrode assays have further leveraged organoids’ ability to replicate neural networks, enabling in-depth studies of neurotoxic effects (Smirnova et al., 2023). These findings underscore the potential of brain organoids in advancing neurotoxicity research and drug safety evaluation. 631.

### Brain–computer interfaces

Research has demonstrated the similarity between cell types in 6-month-old brain organoids derived from patient iPSC lines and those found in human brains, identified through protein markers (Kathuria et al., 2020). Functional assessment, such as electrophysiological activity, further highlights their potential. By 12 months, brain organoids exhibit extensive electrophysiological activity, axonal myelination, and gene expression linked to memory and learning, making them a promising platform for advanced applications.

Notably, brain organoids are being explored for integration with artificial intelligence (AI) through bioengineering techniques like microfluidics and biosensing. Cai et al. (2023) developed Brainoware, a living AI system leveraging the computational capabilities of 3D biological neural networks within brain organoids. This innovation exemplifies how brain organoids can address AI bottlenecks by interfacing with computers and sensors to process complex sensory inputs and outputs, a field termed organoid intelligence (Saglam-Metiner et al., 2019). These advancements highlight the potential of brain organoids as a bridge between biological systems and computational technologies.

### Tissue engineering and BioMEMS applications

Humanized brain organoids can be bioengineered through techniques such as scaffolding, 3D bioprinting, and organ-on-chip systems. Scaffolds, constructed from biomaterials like agarose, Matrigel, collagen (natural) or PEG, PEO, and PVA (synthetic), support cell growth into functional tissues. Advanced 3D bioprinting enables the integration of live cells and biomaterials to construct tissues, offering a bottom-up approach to replicate living physiology (Saglam-Metiner et al., 2024). Microfluidic devices, or “organ-on-chips,” mimic organ-specific functions, facilitating studies on vasculature, brain structure, and circulation. When paired with organoids, these systems incorporate sensors for applications such as neurotoxicity studies, neural circuit activity monitoring, region-specific disease modeling, drug development, and personalized medicine (Tambalo and Lodato, 2020; Spitz et al., 2022; Miccoli et al., 2018). The current cerebral organoid models are culture for 180 days and then used for studies, however with the advancements in techniques such as specialized culturing plates and microfluidic devices, it is possible to now grow these models for 1 year. The vascularization incorporated using either microfluidics or endothelial organoids, helps in making long term culturing possible. Together, these bioengineering approaches enhance the physiological relevance of brain organoids, advancing their potential in research and therapeutics.

### CRISPR technology

iPSCs-derived brain organoids have emerged as a powerful model for studying the intricate cellular diversity and developmental processes of the human brain. These organoids provide a physiologically relevant platform for investigating neurodevelopmental disorders, offering insights into disease mechanisms at a cellular and molecular level. The integration of CRISPR-based gene editing has further expanded the potential of brain organoid research by enabling precise genetic modifications, including gene knockouts and targeted mutations. This approach allows researchers to examine the functional consequences of specific genetic alterations on key neurodevelopmental processes such as synaptic function, neurotransmitter signaling, and neuronal differentiation (Kampmann, 2020).

Moreover, CRISPR technology facilitates large-scale genetic screening in brain organoids derived from iPSCs of patients with neuropsychiatric disorders such as SCZ (Zhang et al., 2023). By employing CRISPR libraries, researchers can systematically target and validate candidate genes implicated in disease pathology. Additionally, introducing patient-specific genetic mutations in ASD-derived brain organoids enables the study of neuronal connectivity defects, providing critical insights into the molecular underpinnings of these conditions (Wang et al., 2017). The convergence of brain organoid technology and CRISPR-based genome editing represents a transformative approach for elucidating disease mechanisms, identifying therapeutic targets, and advancing precision medicine for neurodevelopmental disorders.

## Discussion

The complexity and subjectivity of neuropsychiatric disorders have driven the development of advanced models to enhance understanding of these conditions. The lack of standardized outcomes and unclear mechanisms underlying these disorders highlight the need for innovative research approaches. This review explores cellular phenotypes associated with neuropsychiatric disorders such as ASD, SCZ, BD, AD, MDD, and PTSD. Traditional animal models often fail to accurately mimic human neuropsychiatric pathophysiology, limiting their translational applicability. To address this, patient-derived iPSCs have gained prominence. iPSCs can differentiate into diverse neuronal cell types and be co-cultured to study alterations in cellular phenotypes. Importantly, they retain the donor’s unique genetic profile, enabling investigation into the interplay between genetic predispositions and environmental factors. However, 2D iPSC models lack the structural complexity needed to fully replicate neuropsychiatric disorders.

To overcome this limitation, 3D brain organoids have emerged as critical tools. These models are created through tissue engineering, 3D cell culture, and neural differentiation, offering higher fidelity in modeling human neuropsychiatric diseases. Methods such as single-cell RNA sequencing, electrophysiology, and immunostaining are commonly employed to analyze these models. Despite their advantages, 3D organoids face challenges such as the absence of vascularization and limited representation of specific brain regions. To address these shortcomings, assembloid models are being developed. These integrate various cell types, such as endothelial cells, within brain organoids to better replicate the dynamic, whole-brain effects of neuropsychiatric diseases. These advancements hold promise for more accurate disease modeling and therapeutic development.

## Clinical perspective

In severe mental illness, the complex interplay between genetic factors and environmental stressors, including infectious agents and associated immune dysregulation, throughout the developmental trajectory is crucial (Van Os et al., 2008; Wahbeh and Avramopoulos, 2021; Lockhart et al., 2018). Many critical risk factors for adult-onset mental illness, such as SCZ and mood disorders, impact the brain during early development. iPSC-derived 3D organoids offer a unique advantage in studying these interactions from a temporary perspective. More importantly, by using iPSC-derived 3D organoids, we may have an opportunity to assess the impact of genetic factors, environmental stressors, and gene-environmental interaction in a circuitry- and context-specific manner. Environmental factors often affect glial cells, which in turn influence neurons, and these complex interactions can be more effectively studied in the organoids (Chagas et al., 2020; Zhuo et al., 2023; Hayes et al., 2022). Finally, such 3D information will open up an opportunity of linking the molecular/cellular information to human brain imaging data, such as fMRI observations.
